# Residues Responsible for the Selectivity of α-Conotoxins for Ac-AChBP or nAChRs

**DOI:** 10.3390/md14100173

**Published:** 2016-10-11

**Authors:** Bo Lin, Shihua Xiang, Mengsen Li

**Affiliations:** 1 Hainan Provincial Key Laboratory of Carcinogenesis and Intervention, Hainan Medical College, Haikou 571199, Hainan, China; linbo_752@163.com; 2 Institution of Tumor, Hainan Medical College, Haikou 570102, Hainan, China; 3 Nebraska Center for Virology, School of Veterinary Medicine and Biological Sciences, University of Nebraska-Lincoln, Lincoln, NE 68583, USA

**Keywords:** α-conotoxins, nAChRs, Ac-AChBP, X-ray structure, model, design

## Abstract

Nicotinic acetylcholine receptors (nAChRs) are targets for developing new drugs to treat severe pain, nicotine addiction, Alzheimer disease, epilepsy, etc. α-Conotoxins are biologically and chemically diverse. With 12–19 residues and two disulfides, they can be specifically selected for different nAChRs. Acetylcholine-binding proteins from *Aplysia californica* (Ac-AChBP) are homologous to the ligand-binding domains of nAChRs and pharmacologically similar. X-ray structures of the α-conotoxin in complex with Ac-AChBP in addition to computer modeling have helped to determine the binding site of the important residues of α-conotoxin and its affinity for nAChR subtypes. Here, we present the various α-conotoxin residues that are selective for Ac-AChBP or nAChRs by comparing the structures of α-conotoxins in complex with Ac-AChBP and by modeling α-conotoxins in complex with nAChRs. The knowledge of these binding sites will assist in the discovery and design of more potent and selective α-conotoxins as drug leads.

## 1. Introduction

Nicotinic acetylcholine receptors (nAChRs) are members of ligand-gated ion channels; they respond to the neurotransmitters acetylcholine and choline. Consequentially, nAChRs are drug targets for the treatment of severe pain, cognitive decline, epilepsy, and nicotine addiction [1,2,3,4,5]. α-Conotoxins, which come from sea *Conus* snails, are important tool for studying nAChRs. They are also used as pharmacological probes and as potential therapeutic agents for the treatment of severe pain [6,7,8,9,10,11,12]. α-Conotoxins are diverse and target a variety of nAChR subtypes. Examples include: α-conotoxins GI, SI, and SIA selectively block *Torpedo* nAChR [13,14,15]; α-conotoxins MII and PeIA block rα3β2- and rα6-containing nAChRs [16,17,18]; Vc1.1 and RgIA target the rα9α10 (rat α9α10) nAChR subtypes [19,20,21,22]; ImI targets hα7 (human α7) and hα3β2 nAChRs [23]; and TxIA (A10L) has an affinity for the rα3β2 and h(r)α7 (human and rat α7) subtype receptors [24].

The residues of α-conotoxins are important for determining nAChR subtypes selectivity and potency. For instance, the A10L mutation in α-conotoxin PnIA causes a switch in selectivity from the rα3β2 to the rα7 nAChR [25,26]. Arg-5 in α-conotoxin TxIA is an efficient blocker of rα3β2 nAChR [24]; Arg-7 and Trp-10 in α-conotoxins ImI and ImII were shown to have high affinity for hα7 nAChR [23,27]; Arg-7 in α-conotoxins RgIA and Vc1.1 affected their binding to rα9α10 nAChR [28,29,30,31]; His-5 in several α-conotoxins (GIC, PeIA, OmIA) act as key residues that bind with different neuronal nAChRs [32,33,34,35]; and the positively charged residues Lys and Arg in the C-terminal region increase 3/5 α-conotoxins GI, SI, and SIA, targeting the *Torpedo* nAChR [13,15]. α-Conotoxins consist of two loops: loop 1 residues are more similar and conserved, loop 2 residues are more variable and not conserved. In general, the loop 2 residues are required for selectivity, and the loop 1 residues are crucial for binding (irrespective of the residues, which are most selective) [19]. The residues of α-conotoxin replacements leading to increased potency and selectivity to the receptors are shown in Table 1.

Since α-conotoxins and variants have different potency and selectivity, it is important to understand the characteristics at the molecular level to facilitate discovery and design of potent α-conotoxin analogs [3,41].

**Figure 1:**
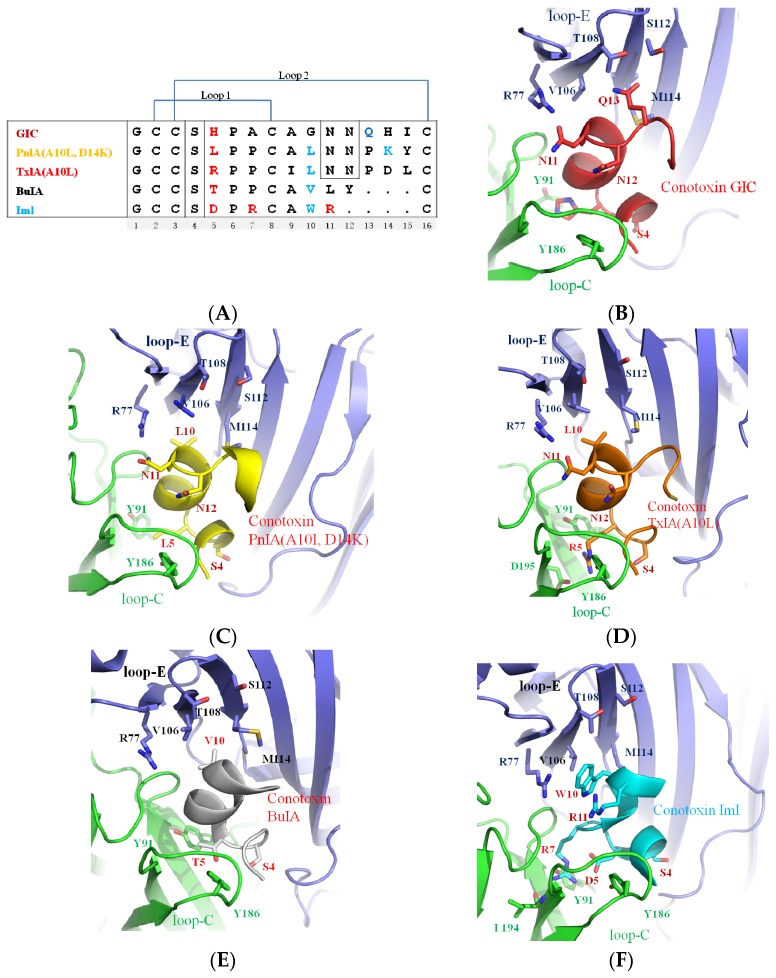
Different crystal structures of α-conotoxins in complex with Ac-AChBP. (**A**) Sequence alignment of α-conotoxins GIC, PnIA (A10L, D14K), TxIA (A10L), BuIA, and ImI; red residues indicate the nonconserved residues selective for the acetylcholine-binding protein from *Aplysia californica* (Ac-AChBP) principal side; blue residues indicate the nonconserved residues selective for the Ac-AChBP complementary side [24,45,47,48]; (**B**) the crystal structure of α-conotoxin GIC in complex with Ac-AChBP (PDB: 5CO5) [47]; (**C**) α-conotoxin PnIA (A10L, D14K) (PDB: 2BR8) [36]; (**D**) α-conotoxin TxIA (A10L) (PDB: 2UZ6) [24]; (**E**) α-conotoxin BuIA [48] (PDB: 4EZ1); and (**F**) α-conotoxin ImI in complex with Ac-AChBP (PDB: 2C9T) [45].

Acetylcholine-binding protein from *Aplysia californica* (Ac-AChBP) is a homologous ligand-binding domain of nAChRs [1,42,43,44]. X-ray structures of α-conotoxins in complex with Ac-AChBP generated new insights into the spatial structure of α-conotoxin interaction with particular nAChRs. The information allowed us to determine the important amino acid residues for binding and selectivity. Currently, five important structures of α-conotoxins in complex with Ac-AChBP have been solved. They are the PnIA variant (PDB: 2BR8) [36], ImI (PDB: 2C9T, 2BYP) [45,46], the TxIA variant (PDB: 2UZ6) [24], BuIA (PDB: 4EZ1), and GIC (PDB: 5CO5) [47]. As shown in Figure 1, the five α-conotoxins in complex with Ac-AChBP structures show a similar spatial structure and nearby binding loop C of the Ac-AChBP; however, the α-conotoxin sequences differ, so they have various binding patterns. For instance, TxIA (A10L) shows a 20° backbone tilt relative to other conotoxin complexes [24].

From the crystal structure of five α-conotoxins in complex with Ac-AChBP, it was found that the five α-conotoxins bound to a similar position of the nearby loop C of the Ac-AChBP, and the binding pattern is also similar. The structures of α-conotoxins are largely determined by the conserved disulfide connectivity between CysI–CysIII and CysII–CysIV [47,49]. The mutations of α-conotoxin, which were observed in the Ala substitutions (no change of the disulfide connectivity), showed no major changes in secondary structure (except Pro residue) [19,38]; the α-conotoxin mutants have a well-defined helix that is similar to native conotoxins. From the structure of α-conotoxins in complex with Ac-AChBP, and as shown by many experiments, it is assumed that the α-conotoxin mutants were binding at a similar site of the receptor and displaying binding patterns similar to native conotoxins [47,49]. Most conotoxins share a common folding structure that is not expected to change upon binding, therefore “rigid” docking can be performed to determine the residues responsible for α-conotoxin’s binding and selectivity for nAChRs [41,50,51,52].

The structure of conotoxins bound by nAChRs is similar to the crystal structure of the conotoxins in complex with Ac-AChBP [36]. For instance, the backbone tilt of conotoxin PnIA (A10L D14K) bound by hα7 nAChR is similar to the crystal structure of PnIA (A10L D14K) in complex with Ac-AChBP (PDB: 2BR8) [36]. This is favorable for using computer models to design and discover new potent α-conotoxins [2,3,51,52,53].

Here, we review the data of the co-crystal structure of α-conotoxins in complex with Ac-AChBP and some of the computer models of α-conotoxins selective for nAChRs, which will help to discover and design α-conotoxin analogs with higher selectivity for nAChRs.

## 2. α-Conotoxin Residue-Binding and Selectivity for Ac-AChBP

### 2.1. Asn-11, Asn-12 Residues of PnIA (A10L D14K), TxIA (A10L), and GIC Are the Key Residues for Binding to Ac-AChBP

**Figure 2:**
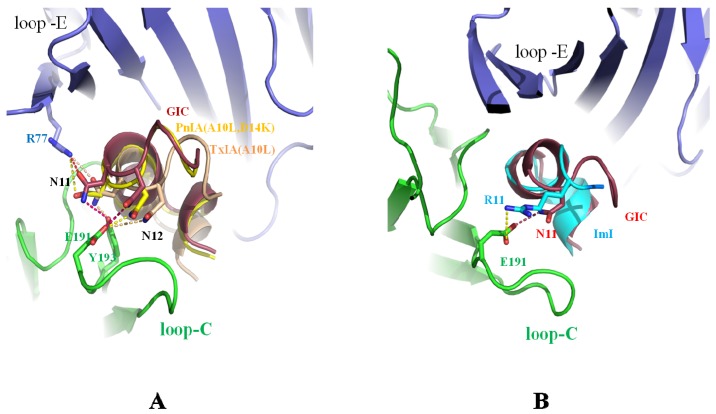
Comparison of different α-conotoxins Asn-11 and Asn-12 bound by Ac-AChBP. (**A**) Asn-11 and Asn-12 of PnIA (A10L D14K) (PDB: 2BR8) [36], TxIA (A10L) (PDB: 2UZ6) [24], and GIC (PDB: 5CO5) [47] positions are similar and form hydrogen bonds with the principal side Glu-191 and Tyr-91 and the complementary side Arg-77 of Ac-AChBP; (**B**) the position of Arg-11 in ImI (PDB: 2C9T) [45] is similar to Asn-11 of GIC, which also contacts the Ac-AChBP principal side residue Glu-191.

Asn-11 and Asn-12 are conserved in many α-conotoxins (shown in Figure 1A) [2]. From the crystal structure of α-conotoxins in complex with Ac-AChBP, it was found that Asn-11 and Asn-12 mainly bind to loop C of Ac-AChBP. A comparison of the Asn-11 and Asn-12 residues of the α-conotoxins in the crystal structure is shown in Figure 2. In Figure 2A, Asn-11 and Asn-12 of PnIA (A10L D14K), TxIA (A10L) and GIC form a hydrogen bond with the principal side (“+”-side, indicated in green, the residue-binding ligand is near loop C) [36] residues, Glu-191 and Tyr-91, and with the complementary side (“−“-side, indicated in blue, the residue-binding ligand is near loop E) [36,51] residues Arg-77. Mutating the residues of Asn-11 and Asn-12 to Ala resulted in the loss of affinity for Ac-AChBP [47]. Arg-11 of ImI is also a key residue for binding to Ac-AChBP. As shown in Figure 2B, the Arg-11 position of ImI is similar to the Asn-11 of GIC, which contacts the Ac-AChBP principal residue Glu-191 [45].

The function of the Asn-11 and Asn-12 residues was also demonstrated by the α-conotoxin PnIA (A10L) truncation assay [54]. PnIA (A10L) loop 2 truncation indicated that removal of the residues (Pro-13, Asp-14, Tyr-15), after Asn-12 did not much affect PnIA (A10L) binding to rα7, but when truncated to Asn-12, the PnIA (A10L) lost the activity to rα7 and acetylcholine-binding protein from *Lymnaea stagnalis* (Ls-AChBP). The sequence length of α-conotoxin ImI is similar to that of PnIA (A10L) as the removal of the residues (Asn-12, Pro-13, Asp-14, Tyr-15), but it has higher affinity to rα7 nAChR. The reason is that the residues Asp-5 and Arg-11 of ImI and the residues Asn-11 and Asn-12 of PnIA (A10L) have similar positions, respectively. Within the binding pocket of rα7, they can form hydrogen bonds with rα7 residues Glu-191, Tyr-193, and Tyr-91. So, ImI activity is actually similar to the full-length PnIA (A10L) [54].

### 2.2. His-5 of GIC, Arg-5 of TxIA (A10L), and Arg-7 of ImI Are the Important Residues Responsible for α-Conotoxin’s Selectivity for the Ac-AChBP Principal Side

**Figure 3:**
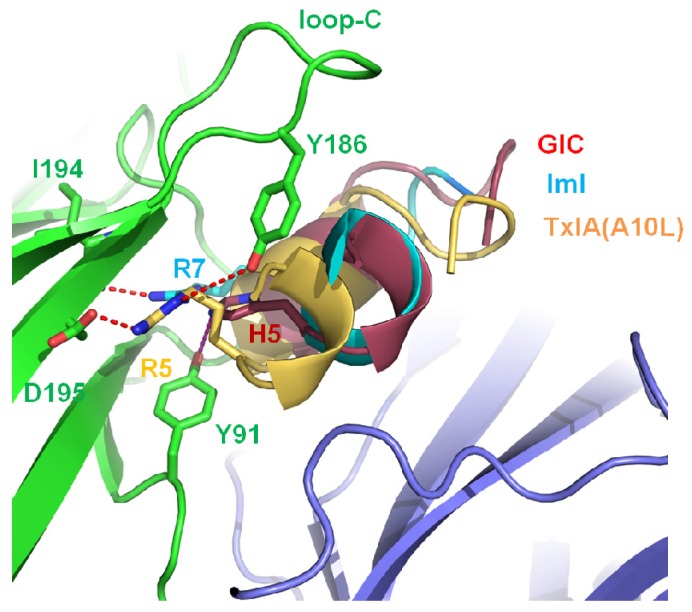
Comparison of His-5 of GIC (PDB: 5CO5) [47], Arg-5 of TxIA (A10L) (PDB: 2UZ6) [24], and Arg-7 of ImI bound by the Ac-AChBP principal side. His-5 of GIC contacts Tyr-91 and Tyr-186 of Ac-AChBP. Arg-5 of TxIA (A10L) protrudes into the principal binding site and contacts Asp-195 and Tyr-186. Arg-7 of ImI (PDB: 2C9T) [45] contacts Tyr-91, Tyr-186, and IIe-194 of Ac-AChBP.

His-5 in α-conotoxin GIC acts as a key residue for contacting with the hα3β2 nAChRs [47]; Arg-5 acts as a key residue, binding with rα3β2 nAChR [24]; and Arg-7 is a key residue of α-conotoxin ImI for its high affinity to hα7 nAChR [45]. Comparison of the crystal structures is shown in Figure 3. His-5 of GIC, Arg-5 of TxIA (A10L), and Arg-7 of ImI reside in similar positions. His-5 of GIC contacts Tyr-91 and Tyr-186; Arg-5 of TxIA (A10L) protrudes into the principal binding site and contacts Asp-195 and Tyr-186; and Arg-7 of ImI is also located in a similar position and contacts Tyr-91, Trp-145, IIe-194, and Asp-195. Mutation of Arg-7 to Ala in ImI resulted in a loss of affinity for Ac-AChBP [24,45].

### 2.3. Ser-4 of GIC, PnIA (A10L D14K), and ImI Are Key Residues for Interaction with the Ac-AChBP Complementary Side

**Figure 4:**
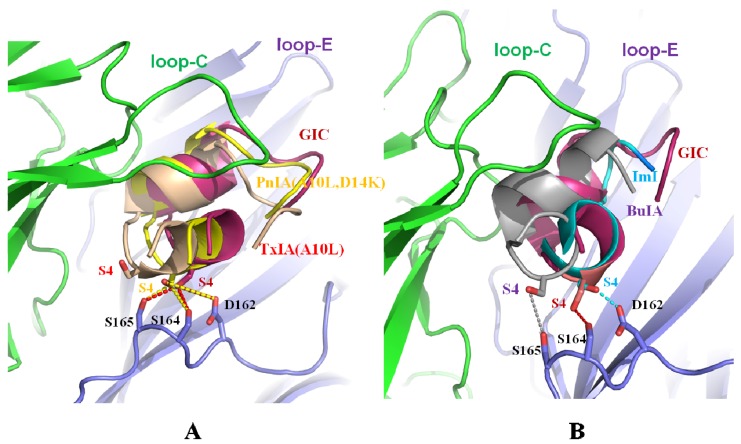
Comparison of different α-conotoxins with Ser-4 bound by the Ac-AChBP complementary side. (**A**) Ser-4 of GIC (PDB: 5CO5) [47] makes hydrogen bonds with Asp-162, Ser-164, or Ser-165. Ser-4 of PnIA (A10L D14K) (PDB: 2BR8) [36] is located in a position similar to that of GIC and forms hydrogen bonds with the three residues. Ser-4 of α-conotoxin TxIA (A10L) (PDB: 2UZ6) [24], with a 20° rotating backbone tilt, makes only weak contact with the Ac-AChBP complementary residue Ser-165; (**B**) Ser-4 of ImI (PDB: 2C9T) [45] is located on the right side of Ser-4 of GIC and contacts Asp-162; Ser-4 of BulA [48] (PDB: 4EZ1) is located on the left side of Ser-4 of GIC and forms a hydrogen bond with Ser-165.

Ser-4 is highly conserved in many α-conotoxins (shown in Figure 1A), which usually binds to the complementary side of Ac-AChBP. Comparison of Ser-4 in the crystal structure of different α-conotoxins is shown in Figure 4. In Figure 4A, it is shown that Ser-4 of GIC and PnIA (A10L D14K) are key residues for binding to the Ac-AChBP complementary side residues Asp-162, Ser-164, and Ser-165. Ser-4 of α-conotoxin TxIA (A10L), with a 20° backbone tilt rotation, makes only weak contact with Ser-165 of the Ac-AChBP complementary side [24,36,47].

Figure 4B shows Ser-4 of ImI located on the right side of Ser-4 of GIC and forms a hydrogen bond with Asp-162. Ser-4 of BulA is located on the left side of Ser-4 of GIC and forms a hydrogen bond with Ser-165 of the Ac-AChBP complementary side [36,47].

### 2.4. Leu-10 of PnIA (A10L D14K) and TxIA (A10L), Gln-13 of GIC, and Trp-10 of ImI Are Important Residues Responsible for α-Conotoxin’s Selectivity for the Ac-AChBP Complementary Side

**Figure 5:**
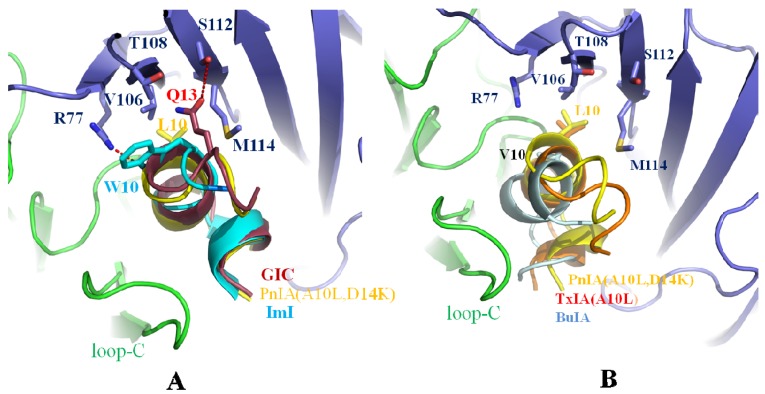
Comparison of different α-conotoxins bound by the Ac-AChBP complementary side pocket consisting of Arg-77, Val-106, Thr-108, Ser-112, and Met-114. (**A**) Leu-10 of PnIA (A10L D14K), Gln-13 of GIC (PDB: 5CO5) [47], and W10 of ImI (PDB: 2C9T) [45] reside in the pocket. Gln-13 of GIC forms a hydrogen bond with Ser-112 of Ac-AChBP [47], but W10 of ImI makes a hydrogen bond with Arg-77 of Ac-AChBP [45]; (**B**) Leu-10 of TxIA (A10L) (PDB: 2UZ6) [24] and Val-10 of BuIA [48] (PDB: 4EZ1) reside in positions similar to Leu-10 of PnIA (A10L D14K), but Val-10 of BuIA forms fewer contacts due to the shorter length of its side-chain compared to Leu-10 and results in a decreased affinity to Ac-AChBP.

Leu-10 in α-conotoxin PnIA (A10L D14K) selects for h(r)α7 nAChRs. Trp-10 of ImI has affinity for h(r)α7 nAChRs. Gln-13 of GIC is an important residue for affinity for hα3β2. The co-crystal comparison is shown in Figure 5. In Figure 5A, Leu-10 of PnIA (A10L D14K), Tyr-10 of ImI, and Gln-13 of GIC reside in a pocket consisting of Arg-77, Val-106, Thr-108, Ser-112, and Met-114 of the complementary Ac-AChBP. Leu-10 of PnIA (A10L D14K) and Gln-13 of GIC serve as anchors and enhance the affinity for the Ac-AChBP complementary side [36,45,47].

Tyr-10 of ImI is also located in the pocket consisting of Arg-77, Val-106, Thr-108, Ser-112, and Met-114. Importantly, the substitution of Val-106 by Arg-106 in Ls-AChBP induces a steric clash with Tyr-10 of ImI, hence the ImI decreases 14,000-fold binding affinity to Ls-AChBP compared with Ac-AChBP [3,45].

Figure 5B shows that Leu-10 of TxIA (A10L) and Val-10 of BuIA [49] reside in positions similar to Leu-10 of PnIA (A10L D14K) and make contact with Arg-77, Val-106, Thr-108, Ser-112, and Met-114 of the Ac-AChBP complementary side, but Val-10 of BuIA forms fewer contacts due to the shorter length of its side-chain compared to Leu-10, resulting in a lower affinity to Ac-AChBP than PnIA (A10L D14K) [24].

## 3. α-Conotoxin Residues Selective for nAChRs

**Figure 6:**
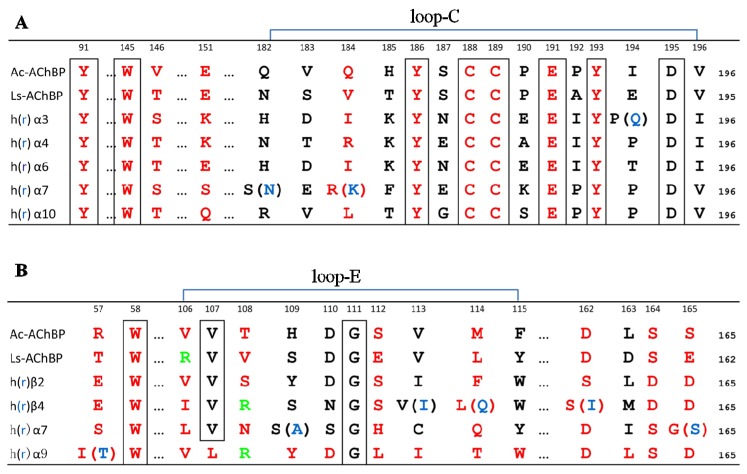
Primary sequence alignment of Ac-AChBP, acetylcholine-binding protein from *Lymnaea stagnalis* (Ls-AChBP), h(r)α3, h(r)α4, h(r)α6, h(r)α7, h(r)α10, h(r) β2, h(r) β4, and h(r) α9 residues forming the binding sites to accommodate α-conotoxins. (**A**) The residues forming the binding sites on the principal side; (**B**) the residues forming the binding sites on the complementary side.

From the α-conotoxins complex with Ac-AChBP structure, and as shown in many experiments, it is assumed that α-conotoxin binding sites of nAChRs are similar to the binding sites of Ac-AChBP [1,36,49]. Sequence alignment of Ac-AChBP, Ls-AChBP, h(r)α3, h(r)α4, h(r)α6, h(r)α7, and h(r)α10 residues forming binding sites on the principal side are shown in Figure 6A. The residues forming binding sites on the complementary side are shown in Figure 6B. In Figure 6, it is shown that different nAChRs have different residues in the binding site, which could affect the conotoxin’s affinity. For example, the Ile-57 (number 59 in that study [31]) of hα9 was replaced by Thr-57 in rα9 (Figure 6B) result in α-conotoxin Vc1.1 increased affinity 10 folds for the rat receptor [31]. Modelling conotoxins binding to nAChRs could determine the key residues of α-conotoxins for a higher affinity to nAChRs [18,31,49].

### 3.1. Residues of α-Conotoxin GIC Selective for hα3β2

**Figure 7:**
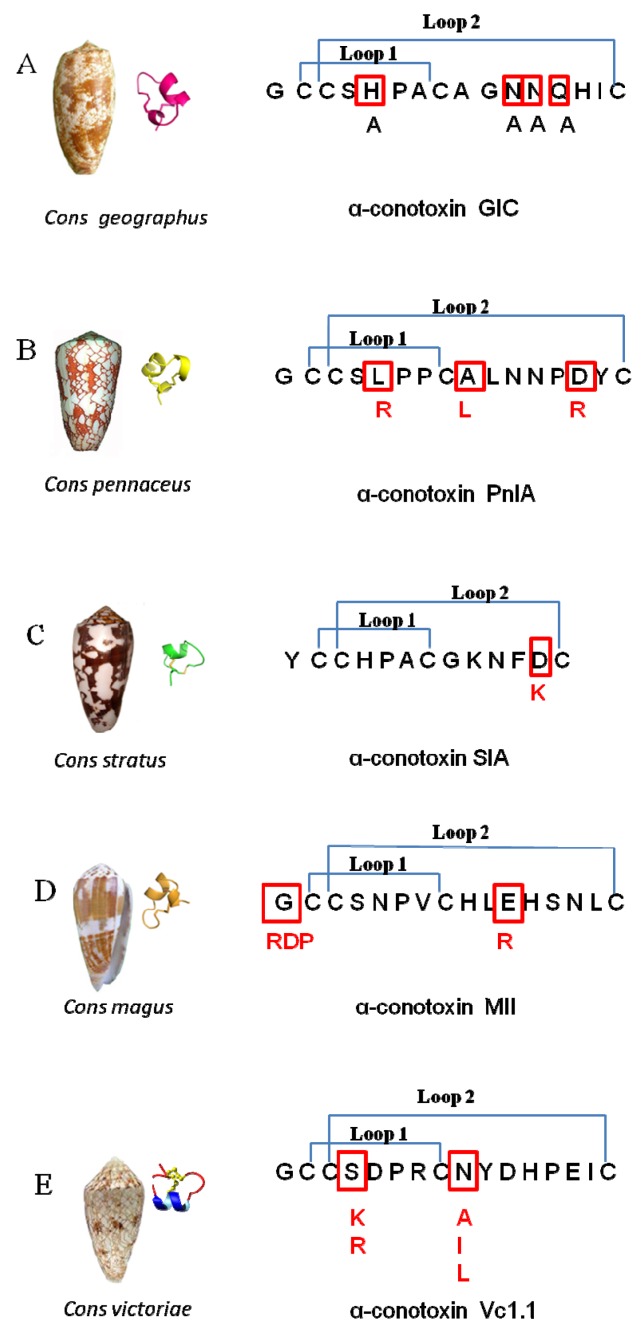
Residues of different α-conotoxins affect selectivity for nicotinic acetylcholine receptors (nAChRs). (**A**) The residues of mutant GIC (H5A), (N11A), (N12A), and (Q13A) affect its selectivity for hα3β2; (**B**) the residues of mutant PnIA (L5R, A10L, D14R) affect its selectivity for hα7; (**C**) the residues of mutant SIA (D12K) affect its selectivity for *Torpedo* nAChR; (**D**) the residues of mutant MII (G1RDP) and (E11R) affect its selectivity for rα6β2 or rα3β2 nAChR; (**E**) the residues of mutant Vc1.1 (S4K, R) and (N9A, I, L) affect its selectivity for h(r)α9rα10 nAChRs.

As we have mentioned before, Gln-13 and His-5 of α-conotoxin GIC have high affinity for hα3β2. By docking α-conotoxin GIC to hα3β2, it was found that the GIC/hα3β2 complex was similar to the GIC/Ac-AChBP crystal structure. Mutation of His-5, Asn-11, and Asn-12 to Ala in α-conotoxin GIC (Figure 7A) caused a loss of affinity for Ac-AChBP. The mutations also lost affinity for hα3β2. Only the Gln13-Ala substitution had the least influence on GIC activity in hα3β2 or Ac-AChBP. From the GIC/hα3β2 complex model, it was found that Gln-13-Ala substitution had little effect, and it fit well with the receptor. Gln-13 or Ala-13 of GIC also fit well with the hα3β2 receptor, but Gln-13 of GIC caused a steric clash with Arg-108 in the hβ4 complementary binding side (Figure 6B) and lost affinity to hα3β4 [35,47].

### 3.2. Residues of α-Conotoxin PnIA Selective for hα7

As previously mentioned, A10L in PnIA (A10L) has higher affinity to Ac-AChBP and hα7 nAChR [36]. The docking study found that Leu-10 of PnIA (A10L) interacts with the hα7 nAChR hydrophobic pocket, which is composed of the residues Leu-106, Asn-108, His-112, and Gln-114 in the complementary side (Figure 6B). Consequently, A10L mutation could extend the hydrophobic regions and increase the binding to the hydrophobic pocket of the receptor [3,36,37].

PnIA mutations (Figure 7B) PnIA (L5R, A10L, D14R) display a 10-fold higher affinity for the hα7 nAChR compared to that of PnIA (A10L) [37]. The reason could be explained by Leu-5 of the PnIA being in the vicinity of hα7 residues Asp-195, Tyr-193 (loop C), and Tyr-91. In the L5R mutation, the Arg-5 of the PnIA could form hydrogen bonds with those residues of hα7. Asp-14 of the PnIA is in the vicinity of hα7 residues His-114, Asn-110, and Lys75. The D14K mutation in PnIA (A10L) does not have much effect on its affinity to hα7 [36], but PnIA (L5R, A10L, D14R) shows high affinity to hα7; this may be due to the fact that the Arg-14 forms hydrogen bonds with Asn-110 residues of hα7 nAChRs [37].

### 3.3. Residues of α-Conotoxins SIA Selective for Torpedo nAChR

The PnIA (A10L D14K) mutation shows higher affinity for Ls-AChBP. Modeling studies found that the Lys-14 residue of PnIA (A10L D14K) forms a salt bridge with Glu-110 of the Ls-AChBP complementary site [36]. Kasheverov et al. found that adding positively charged residues (Lys or Arg) in the C-terminal region of α-conotoxins increased binding affinity to some nAChRs [15]. For example, the α-conotoxin SIA (D12K) mutation (Figure 7C) showed higher affinity of *Torpedo* nAChRs. The reason could be similar to the D14K mutation in PnIA (A10L) which gained a higher affinity for Ls-AChBP [15]. Its Lys-12 in SIA (D12K) formed a salt bridge with Glu-57 of the *Torpedo* nAChRγ subunit and increased binding affinity to it [15].

### 3.4. Residues of α-Conotoxins MII Selective for rα6β2 nAChRs

Gotti et al. discovered that the α**-**conotoxin MII selectively blocked rα6β2 nAChRs [40], Gly-1 of MII was mutated to three amino acids RDP (arginine (R), aspartic acid (D), and proline (P)) (Figure 7D), and the N-terminal amino acid tail motif RDP of MII increased affinity for the rα6β2 receptor by 13-fold. Modeling studies found that Arg-1 of the RDP motif makes hydrogen bonds with Asp-166 and Asp-167 of the β2 subunit [40]. 

**Figure 8:**
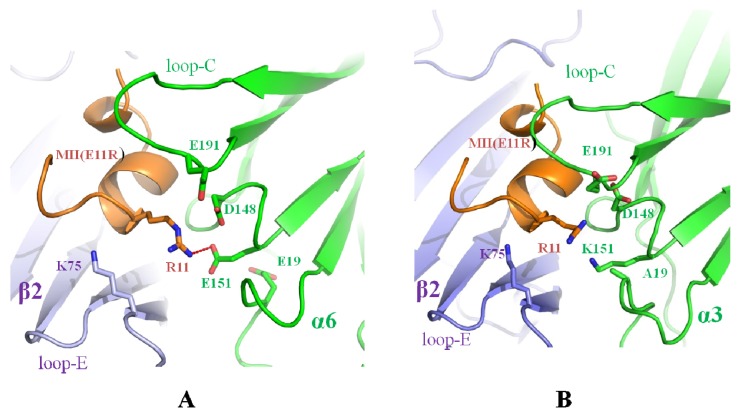
Structure basis for the selectivity of MII (E11R) with rα6β2 vs. rα3β2 nAChRs. (**A**) The pocket in rα6 is able to accommodate Arg-11 of MII (E11R), and Arg-11 forms a hydrogen bond with Glu-151 in the α6β2/MII (E11R) model; (**B**) Lys-151 in the pocket of rα3 is unfavorable for Arg-11 of MII (E11R) binding and then lost affinity to rα3β2 compared with the rα6β2 nAChRs.

Mutation of the conotoxin MII to MII (E11R) with a highly positively charged residue Arg-11 also favors local contact with the negatively charged area (Glu-19, Asp-148, Glu-151, Glu-191), and Arg-11 makes a hydrogen bond with Glu-151 (Figure 8A). So the MII (E11R) mutation also has affinity to rα6β2. Glu-19 and Glu-151 in the α6 chain are replaced by Ala-19 and Lys-151 in the α3 receptor, thus making the α3 receptor binding site more positively charged and not favorable for Arg-11 of MII(E11R) to be bound by the receptor. Then, the MII (E11R) lost affinity for rα3β2 (Figure 8B). So MII (E11R) could be selective for rα6β2 vs. rα3β2 [40].

### 3.5. Residues of α-Conotoxins Vc1.1 Selective for α9α10 nAChR

Vc1.1 is a conotoxin from *Conus Victoriae* (Figure 7E). Mutations of Vc1.1 residues 5–7 and 11–15 to Ala, Lys, or Asp led to a significant decrease of functional activity at the α9α10 nAChR. The reason is that all these mutations can cause a disruption in the native structure that result in all the peptides unable to bind to the receptor, or alternatively affecting the specific amino acid interactions [19].

In contrast, substitutions at two other positions, Ser-4 and Asn-9, could improve the functional activity of Vc1.1 at the α9α10 nAChR. Replacement of Ser-4 by a positive residue (Arg or Lys), is more favorable for the potency at the rα9α10 and hα9rα10 nAChRs, but not for other receptors [19]. Vc1.1 mainly binds to α9α10 nAChR at the α10α9 pocket (α10 is principle site (+), and α9 is complementary site (−)). The receptor residues involved in interactions with Ser-4 of Vc1.1 are Asp-169, Asp-166, and Thr-32 of α9(−) [31]. Replacement of Ser-4 by Arg or Lys in Vc1.1, may favor the positive residue binding to those three residues, so increases potency at the α9α10 nAChR.

The receptor residues involved in interactions with Asn-9 of Vc1.1 are Gln-34, Arg-57, Thr-59, Thr-117 of rα9(−).The Thr-59 of rα9(−) is replaced by Ile-59 in hα9(−) (the number in that study). Asn-9, when replaced with a hydrophobic residue (Ala, Ile, Leu), will form more hydrophobic regions, so affinity to the receptors increased and resulted in more favorable binding potency at the rα9α10 and hα9rα10 nAChRs [19].

From those findings above, it could be suggested that the molecular structure combined with technology such as scanning mutagenesis, electrophysiology, etc. [18,19], not only helped us find the residues of α-conotoxins for various nAChR subtype selectivity, but also helped us design α-conotoxin analogs of higher potency and selectivity for drug application [41,53,54,55].

For example, the α-conotoxin Vc1.1 potent variants such as N9A, N9I, N9L and S4R, which are highly selective for rα9α10, were first discovered by scanning mutagenesis by Craik et al. [19]. Using different computational strategies for modeling α-conotoxin Vc1.1 binding to nAChRs further determined key residues and binding sites that helped with the design of the new selective analog Vc1.1 (N9W) [31]. It also helps in the design of stable disulfide-deleted mutant of cyclic α-conotoxin Vc1.1 (C2H C8F) for oral drug application to treatment of neuropathic pain [56,57].

## 4. Conclusions

The diversity of α-conotoxins makes them not only excellent pharmacological probes for the dissection of structural features and functioning of nAChRs but also provides drug leads; some α-conotoxins are currently undergoing preclinical evaluation for the treatment of chronic and neuropathic pain. X-ray structures of α-conotoxins in complex with Ac-AChBP provide high-resolution information of these amino acid interactions and binding patterns. Based on co-crystal structures, computer models further revealed α-conotoxin residue selectivity for different nAChR subtypes and helped with the design of potent analogs. So, many potent α-conotoxins have been discovered by using the molecular structure of α-conotoxins selective for nAChRs, and here we only list some representatives. Currently, the optimization of drugs usually depends on animal models, but the drugs intended for human use could be misleading, as large activity differences can arise between binding site residues displaying some differences. A structural base provides insight to the important residues for binding to receptors and aids in the design of α-conotoxin analogs that are more potent for accurate treatment.

## Figures and Tables

**Table 1:** The residues of α-conotoxin replacements leading to increased potency and selectivity to the receptors.

α-Conotoxins	Potent Variant	Targed AChBPs or nAChRs	Increasing Affinity
PnIA	PnIA (A10L)	h(r)α7	~10-fold [26,36]
	PnIA (L5R A10L)	rα3β2	20-fold [24]
	PnIA (A10L D14K)	Ls-AChBP	10-fold [36]
	PnIA (L5R, A10L, D14R)	hα7	21-fold [37]
	PnIA (L5H)	Ac-AChBP	18-fold [37]
TxIA	TxIA (A10L)	rα3β2	~1.5-fold [24]
	TxIA (A10L)	rα7	10-fold [24]
SIA	SIA (D12S)	*Torpedo*	27-fold for high affinity site [15]
	SIA (D12K)	*Torpedo*	35-fold for high affinity site [15]
GI	GI (S12R)	*Torpedo*	3-fold for high affinity site [15]
	GI (S12K)	*Torpedo*	5-fold for high affinity site [15]
SI	SI (S12R)	*Torpedo*	4-fold for high affinity site [15]
GID	GID (γ4E)	rα4β2	1-fold [38,39]
PeIA	PeIA (H5N)	rα6/α3β2β3	6-fold [18]
	PeIA (A7V)	rα6/α3β2β3	2-fold [18]
	PeIA (S9R)	rα6/α3β2β3	7-fold [18]
	PeIA (A7V, S9H, V10A, N11R, E14A)	rα6/α3β2β3	~8-fold [18]
	PeIA (S9R)	rα3β2	4-fold [18]
	PeIA (V10L)	rα3β2	5-fold [18]
Vc1.1	Vc1.1 (N9G, A, I, L), (S4R)	rα9α10	~8-fold [19]
	Vc1.1 (N9G, A, I, L), (S4R), (S4K, N9A)	hα9rα10	~25-fold [19]
	Vc1.1 (N9A, I, L)	rα3β2	~50, 20, 2-fold respectively [19]
	Vc1.1 (N9W)	hα9rα10	~30-fold [31]
MII	RDP-MII	rα6β2	13-fold [40]
	RDP-MII (E11R)	rα6β2	13-fold [40]

## References

[1] Van Dijk W.J., Klaassen R.V., Schuurmans M., van der Oost J., Smit A.B., Sixma T.K. (2001). Crystal structure of an ACh-binding protein reveals the ligand-binding domain of nicotinic receptors. Nature.

[2] Kasheverov I.E., Utkin Y.N., Tsetlin V.I. (2009). Naturally occurring and synthetic peptides acting on nicotinic acetylcholine receptors. Curr. Pharm. Des..

[3] Rucktooa P., Smit A.B., Sixma T.K. (2009). Insight in nAChR subtype selectivity from AChBP crystal structures. Biochem. Pharmacol..

[4] Sadigh-Eteghad S., Majdi A., Mahmoudi J., Golzari S.E., Talebi M. (2016). Astrocytic and microglial nicotinic acetylcholine receptors: An overlooked issue in Alzheimer’s disease. J. Neural Transm..

[5] Nemecz A., Prevost M.S., Menny A., Corringer P.J. (2016). Emerging Molecular Mechanisms of Signal Transduction in Pentameric Ligand-Gated Ion Channels. Neuron.

[6] Armishaw C.J., Alewood P.F. (2005). Conotoxins as research tools and drug leads. Curr. Protein Pept. Sci..

[7] Akondi K.B., Muttenthaler M., Dutertre S., Kaas Q., Craik D.J., Lewis R.J., Alewood P.F. (2014). Discovery, synthesis, and structure-activity relationships of conotoxins. Chem. Rev..

[8] Lebbe E.K., Peigneur S., Wijesekara I., Tytgat J. (2014). Conotoxins targeting nicotinic acetylcholine receptors: An overview. Mar. Drugs.

[9] Mir R., Karim S., Kamal M.A., Wilson C.M., Mirza Z. (2016). Conotoxins: Structure, Therapeutic Potential and Pharmacological Applications. Curr. Pharm. Des..

[10] Wu R.J., Wang L., Xiang H. (2015). The Structural Features of α-Conotoxin Specifically Target Different Isoforms of Nicotinic Acetylcholine Receptors. Curr. Top. Med. Chem..

[11] Wang S., Zhao C., Liu Z., Wang X., Liu N., Du W., Dai Q. (2015). Structural and functional characterization of a novel α-conotoxin Mr1.7 from Conus marmoreus targeting neuronal nAChR α3β2, α9α10 and α6/α3β2β3 subtypes. Mar. Drugs.

[12] Olivera B.M., Rivier J., Clark C., Ramilo C.A., Corpuz G.P., Abogadie F.C., Mena E.E., Woodward S.R., Hillyard D.R., Cruz L.J. (1990). Diversity of Conus neuropeptides. Science.

[13] Kasheverov I.E., Chiara D.C., Zhmak M.N., Maslennikov I.V., Pashkov V.S., Arseniev A.S., Utkin Y.N., Cohen J.B., Tsetlin V.I. (2006). α-Conotoxin GI benzoylphenylalanine derivatives. FEBS J..

[14] Groebe D.R., Gray W.R., Abramson S.N. (1997). Determinants involved in the affinity of α-conotoxins GI and SI for the muscle subtype of nicotinic acetylcholine receptors. Biochemistry.

[15] Kasheverov I.E., Zhmak M.N., Vulfius C.A., Gorbacheva E.V., Mordvintsev D.Y., Utkin Y.N., van Elk R., Smit A.B., Tsetlin V.I. (2006). α-Conotoxin analogs with additional positive charge show increased selectivity towards Torpedo californica and some neuronal subtypes of nicotinic acetylcholine receptors. FEBS J..

[16] Quik M., Bordia T., Forno L., McIntosh J. (2004). Loss of α-conotoxinMII- and A85380-sensitive nicotinic receptors in Parkinson’s disease striatum. J. Neurochem..

[17] Cartier G.E., Yoshikami D., Gray W.R., Luo S., Olivera B.M., McIntosh J.M. (1996). A new-conotoxin which targets α3β2 nicotinic acetylcholine receptors. J. Biol. Chem..

[18] Hone A.J., Ruiz M., Scadden M., Christensen S., Gajewiak J., Azam L., McIntosh J.M. (2013). Positional scanning mutagenesis of α-conotoxin PeIA identifies critical residues that confer potency and selectivity for α6/α3β2β3 and α3β2 nicotinic acetylcholine receptors. J. Biol. Chem..

[19] Halai R., Clark R.J., Nevin S.T., Jensen J.E., Adams D.J., Craik D.J. (2009). Scanning mutagenesis of α-conotoxin Vc1.1 reveals residues crucial for activity at the α9α10 nicotinic acetylcholine receptor. J. Biol. Chem..

[20] Halai R., Callaghan B., Daly N.L., Clark R.J., Adams D.J., Craik D.J. (2011). Effects of cyclization on stability, structure, and activity of α-conotoxin RgIA at the α9α10 nicotinic acetylcholine receptor and GABA(B) receptor. J. Med. Chem..

[21] Ellison M., Feng Z.P., Park A.J., Zhang X., Olivera B.M., McIntosh J.M., Norton R.S. (2008). Alpha-RgIA, a novel conotoxin that blocks the α9α10 nAChR: Structure and identification of key receptor-binding residues. J. Mol. Biol..

[22] Pacini A., Micheli L., Maresca M., Branca J.J., McIntosh J.M., Ghelardini C., Di Cesare Mannelli L. (2016). The α9α10 nicotinic receptor antagonist α-conotoxin RgIA prevents neuropathic pain induced by oxaliplatin treatment. Exp. Neurol..

[23] Ellison M., Gao F., Wang H.L., Sine S.M., McIntosh J.M., Olivera B.M. (2004). α-Conotoxins ImI and ImII target distinct regions of the human α7 nicotinic acetylcholine receptor and distinguish human nicotinic receptor subtypes. Biochemistry.

[24] Dutertre S., Ulens C., Büttner R., Fish A., van Elk R., Kendel Y., Hopping G., Alewood P.F., Schroeder C., Nicke A. (2007). AChBP-targeted α-conotoxin correlates distinct binding orientations with nAChR subtype selectivity. EMBO J..

[25] Fainzilber M., Hasson A., Oren R., Burlingame A.L., Gordon D., Spira M.E., Zlotkin E. (1994). New Mollusk-Specific. α-Conotoxins Block Aplysia Neuronal Acetylcholine Receptors. Biochemistry.

[26] Hogg R.C., Miranda L.P., Craik D.J., Lewis R.J., Alewood P.F., Adams D.J. (1999). Single amino acid substitutions in α-conotoxin PnIA shift selectivity for subtypes of the mammalian neuronal nicotinic acetylcholine receptor. J. Biol. Chem..

[27] Ellison M., McIntosh J.M., Olivera B.M. (2003). α-Conotoxins ImI and ImII similar α7 nicotinic receptor antagonists act at different sites. J. Biol. Chem..

[28] Ellison M., Haberlandt C., Gomez-Casati M.E., Watkins M., Elgoyhen A.B., McIntosh J.M., Olivera B.M. (2006). α-RgIA: A novel conotoxin that specifically and potently blocks the α9α10 nAChR. Biochemistry.

[29] Azam L., McIntosh J.M. (2012). Molecular basis for the differential sensitivity of rat and human α9α10 nAChRs to α-conotoxin RgIA. J. Neurochem..

[30] Clark R.J., Fischer H., Nevin S.T., Adams D.J., Craik D.J. (2006). The synthesis, structural characterization, and receptor specificity of the α-conotoxin Vc1.1. J. Biol. Chem..

[31] Yu R., Kompella S.N., Adams D.J., Craik D.J., Kaas Q. (2013). Determination of the α-conotoxin Vc1.1 binding site on the α9α10 nicotinic acetylcholine receptor. J. Med. Chem..

[32] McIntosh J.M., Dowell C., Watkins M., Garrett J.E., Yoshikami D., Olivera B.M. (2002). α-Conotoxin GIC from Conus geographus, a novel peptide antagonist of nicotinic acetylcholine receptors. J. Biol. Chem..

[33] McIntosh J.M., Plazas P.V., Watkins M., Gomez-Casati M.E., Olivera B.M., Elgoyhen A.B. (2005). A novel α-conotoxin, PeIA, cloned from Conus pergrandis, discriminates between rat α9α10 and α7 nicotinic cholinergic receptors. J. Biol. Chem..

[34] Talley T.T., Olivera B.M., Han K.H., Christensen S.B., Dowell C., Tsigelny I., Ho K.Y., Taylor P., McIntosh J.M. (2006). α-Conotoxin OmIA is a potent ligand for the acetylcholine-binding protein as well as α3β2 and α7 nicotinic acetylcholine receptors. J. Biol. Chem..

[35] Chi S., Kim D., Olivera B., McINTOSH J., Han K. (2004). Solution conformation of α-conotoxin GIC, a novel potent antagonist of α3β2 nicotinic acetylcholine receptors. Biochem. J..

[36] Celie P.H., Kasheverov I.E., Mordvintsev D.Y., Hogg R.C., van Nierop P., van Elk R., van Rossum-Fikkert S.E., Zhmak M.N., Bertrand D., Tsetlin V. (2005). Crystal structure of nicotinic acetylcholine receptor homolog AChBP in complex with an α-conotoxin PnIA variant. Nat. Struct. Mol. Biol..

[37] Kasheverov I.E., Zhmak M.N., Khruschov A.Y., Tsetlin V.I. (2011). Design of New α-Conotoxins: From Computer Modeling to Synthesis of Potent Cholinergic Compounds. Mar. Drugs.

[38] Millard E.L., Nevin S.T., Loughnan M.L., Nicke A., Clark R.J., Alewood P.F., Lewis R.J., Adams D.J., Craik D.J., Daly N.L. (2009). Inhibition of neuronal nicotinic acetylcholine receptor subtypes by α-Conotoxin GID and analogues. J. Biol. Chem..

[39] Nicke A., Loughnan M.L., Millard E.L., Alewood P.F., Adams D.J., Daly N.L., Craik D.J., Lewis R.J. (2003). Isolation, structure, and activity of GID, a novel α 4/7-conotoxin with an extended N-terminal sequence. J. Biol. Chem..

[40] Pucci L., Grazioso G., Dallanoce C., Rizzi L., De Micheli C., Clementi F., Bertrand S., Bertrand D., Longhi R., De Amici M., Gotti C. (2011). Engineering of α-conotoxin MII-derived peptides with increased selectivity for native α6β2* nicotinic acetylcholine receptors. FASEB J..

[41] Daly N.L., Craik D.J. (2009). Structural studies of conotoxins. IUBMB Life.

[42] Unwin N. (2005). Refined structure of the nicotinic acetylcholine receptor at 4 Å resolution. J. Mol. Biol..

[43] Tsetlin V., Hucho F. (2009). Nicotinic acetylcholine receptors at atomic resolution. Curr. Opin. Pharmacol..

[44] Buchapudi K., Xu X., Ataian Y., Ji H.-F., Schulte M. (2012). Micromechanical measurement of AChBP binding for label-free drug discovery. Analyst.

[45] Ulens C., Hogg R.C., Celie P.H., Bertrand D., Tsetlin V., Smit A.B., Sixma T.K. (2006). Structural determinants of selective α-conotoxin binding to a nicotinic acetylcholine receptor homolog AChBP. Proc. Natl. Acad. Sci. USA.

[46] Hansen S.B., Sulzenbacher G., Huxford T., Marchot P., Taylor P., Bourne Y. (2005). Structures of Aplysia AChBP complexes with nicotinic agonists and antagonists reveal distinctive binding interfaces and conformations. EMBO J..

[47] Lin B., Xu M., Zhu X., Wu Y., Liu X., Zhangsun D., Hu Y., Xiang S.H., Kasheverov I.E., Tsetlin V.I. (2016). From crystal structure of α-conotoxin GIC in complex with Ac-AChBP to molecular determinants of its high selectivity for α3β2 nAChR. Sci. Rep..

[48] Jin A.H., Brandstaetter H., Nevin S.T., Tan C.C., Clark R.J., Adams D.J., Alewood P.F., Craik D.J., Daly N.L. (2007). Structure of α-conotoxin BuIA: Influences of disulfide connectivity on structural dynamics. BMC Struct. Biol..

[49] Grishin A.A., Cuny H., Hung A., Clark R.J., Brust A., Akondi K., Alewood P.F., Craik D.J., Adams D.J. (2013). Identifying Key Amino Acid Residues That Affect α-Conotoxin AuIB Inhibition of α3β4 Nicotinic Acetylcholine Receptors. J. Biol. Chem..

[50] Millard E.L., Daly N.L., Craik D.J. (2004). Structure-activity relationships of α-conotoxins targeting neuronal nicotinic acetylcholine receptors. Eur. J. Biochem..

[51] Zhangsun D., Zhu X., Wu Y., Hu Y., Kaas Q., Craik D.J., McIntosh J.M., Luo S. (2015). Key residues in the nicotinic acetylcholine receptor β2 subunit contribute to α-conotoxin LvIA binding. J. Biol. Chem..

[52] Luo S., Zhangsun D., Schroeder C.I., Zhu X., Hu Y., Wu Y., Weltzin M.M., Eberhard S., Kaas Q., Craik D.J. (2014). A novel α4/7-conotoxin LvIA from Conus lividus that selectively blocks α3β2 vs. α6/α3β2β3 nicotinic acetylcholine receptors. FASEB J..

[53] Lewis R.J., Dutertre S., Vetter I., Christie M.J. (2012). Conus venom peptide pharmacology. Pharmacol. Rev..

[54] Jin A.H., Daly N.L., Nevin S.T., Wang C.I., Dutertre S., Lewis R.J., Adams D.J., Craik D.J., Alewood P.F. (2008). Molecular engineering of conotoxins: The importance of loop size to α-conotoxin structure and function. J. Med. Chem..

[55] Halai R., Craik D.J. (2009). Conotoxins: Natural product drug leads. Nat. Prod. Rep..

[56] Yu R., Seymour V.A., Berecki G., Jia X., Akcan M., Adams D.J., Kaas Q., Craik D.J. (2015). Less is More: Design of a Highly Stable Disulfide-Deleted Mutant of Analgesic Cyclic α-Conotoxin Vc1.1. Sci. Rep..

[57] Clark R.J., Jensen J., Nevin S.T., Callaghan B.P., Adams D.J., Craik D.J. (2010). The engineering of an orally active conotoxin for the treatment of neuropathic pain. Angew. Chem. Int. Ed. Engl..

